# Experimental studies on comparison of the vector competence of four Italian *Culex pipiens* populations for West Nile virus

**DOI:** 10.1186/s13071-015-1067-z

**Published:** 2015-09-17

**Authors:** Claudia Fortuna, Maria Elena Remoli, Marco Di Luca, Francesco Severini, Luciano Toma, Eleonora Benedetti, Paola Bucci, Fabrizio Montarsi, Giada Minelli, Daniela Boccolini, Roberto Romi, Maria Grazia Ciufolini

**Affiliations:** Unit of Viral diseases and attenuated vaccine, Department of Infectious, Parasitic and Immune-Mediated Diseases Istituto Superiore di Sanità, Rome, Italy; Unit of Vector-borne Diseases and International Health, Department of Infectious, Parasitic and Immune-Mediated Diseases Istituto Superiore di Sanità, Rome, Italy; Istituto Zooprofilattico Sperimentale delle Venezie, Legnaro, PD Italy; National Centre for Epidemiology, Surveillance and Health Promotion, Unit of Statistics Istituto Superiore di Sanità, Rome, Italy

**Keywords:** Vector competence, Infection, West Nile virus, Cx. pipiens, Mosquitoes, Italy

## Abstract

**Background:**

West Nile virus (WNV) is a vector-borne disease responsible for causing epidemics in many areas of the world. The virus is maintained in nature by an enzootic bird-mosquito-bird cycle and occasionally transmitted to other hosts, such as equines and humans. *Culex* species, in particular the ubiquitous species *Culex pipiens* is thought to play a major vector role both in enzootic and epizootic maintenance and transmission of WNV. Introduced in Europe in recent years, since 2008 WNV has been stably circulating mainly in the Northeastern regions of Italy, although sporadic equine and/or human cases, as well as WNV infected *Cx. pipiens* pools, have been recorded in other Italian areas. The scope of our study was to evaluate the potential competence of some Italian populations of *Cx. pipiens* to transmit WNV and to assess their ability for vertical transmission of the virus. For this purpose four Italian populations, from different areas, were experimentally infected.

**Methods:**

After the infectious blood meal, fed females were monitored for 32 days to determine the length of viral extrinsic incubation period. WNV titre of infected mosquitoes was evaluated both by quantitative Real Time PCR and viral titration by Plaque Forming Units/ml (PFU/mL) in VERO cells. Potential Infection, Dissemination, Transmission rates (IR, DR, TR) were assessed by detection of the virus in body, legs plus wings and saliva of the fed females, respectively.

**Results:**

All tested populations were susceptible to the WNV infection. The viral presence in legs and wings demonstrated the ability of WNV to disseminate in the mosquitoes. Viral RNA was detected in the saliva of tested populations. No significant differences in TR values were observed among the four studied populations. The offspring of the *Cx. pipiens* infected females were WNV negative.

**Conclusions:**

Our study addressed an important issue in the knowledge on the complex WNV-vector relationships in Italy, indicating that all Italian *Cx. pipiens* populations tested exhibited vector competence for WNV. Further studies should be performed in order to better clarify the role of other factors (vector density, climatic conditions, reservoir presence etc.) in order to predict where and when WNV outbreaks could occur.

## Background

West Nile virus (WNV, family *Flaviviridae*, genus *Flavivirus*) is an emerging zoonotic arthropod-borne virus (arbovirus) widely distributed throughout the world and with considerable impact on veterinary and human health [1, 2]. The virus is maintained and amplified in nature within an enzootic transmission cycle among birds and ornithophilic mosquitoes [3]. The virus was first isolated in 1937 in Uganda, then sporadic cases and epidemic outbreaks were reported in other African countries and in the Middle-East, mainly involving horses and humans [4]. In 1999 an important WNV outbreak occurred in New York City, while in Europe the virus was only reported up to the end of the 1990’s [5–7]. The largest human outbreaks occurred in Romania in 1996 and Russia in 1999 [8, 9]. Concerning other countries in the EU area, cases of WNV encephalitis were initially recorded in 2000 on horses in Southern France [10] and seropositivity for the WNV was reported for horses, humans and birds in Portugal [11, 12] and Spain [13, 14]. In Spain, the first clinical cases of WNV were reported on horses and humans in 2010 [15]. Since 2003 evidence of WNV circulation among birds has been reported also in the UK [16]. Nevertheless, in Austria, Germany and Czech Republic increasing WNV antibody titres were highlighted between 2009–2010 [17]. In the last years, WNV caused large human epidemics in the Balkan area including Greece and Romania [18, 19]. In Italy, the first WNV outbreak occurred in Tuscany in 1998 involving horses only [20]. No further outbreaks were reported until late summer of 2008 when human cases of WNV disease were reported in the regions of Emilia Romagna, Lombardia and Veneto (North-Eastern Italy). These regions are now considered endemic for this virus. From 2008–2012 WNV foci were also reported in other Italian regions (Sardinia, Sicily, Calabria, Basilicata and Marche), with a total of 73 human cases of confirmed neuro-invasive disease [21–25]. More recently, from June to November 2013, 40 cases of neuro-invasive disease have been reported in Italy with a mortality rate of 17.5 % [26]. Entomological and epidemiological surveillance have highlighted the circulation in Italy of both lineage 1 and 2 [25, 27].

Mosquitoes species belonging to the genus *Culex* are considered important vectors for WNV. In particular *Culex pipiens* is thought to play a major role both in enzootic and epizootic cycles [28–31]. This species comprises two distinct forms, *pipiens* and *molestus*, that differ in several behavioural and ecological characteristics affecting their vector competence [32]. When in sympatry, *molestus* and *pipiens* forms may interbreed generating hybrids that with their intermediate features may act as WNV-bridge vectors for WNV [33, 34].

Currently most of our knowledge of ecology, dynamics and genetic composition of vector populations, come from North America [35, 36]. Conversely, in Europe the distribution and circulation dynamics of WNV are not completely clear. WNV outbreaks occur nearly every year but in different and often widely separated regions. A number of studies have proposed that this pattern is the result of infected migratory birds arriving from Africa and seeding the virus in different areas [37].

The complexity of WNV eco-epidemiologic characteristics, involving different vectors and host species, has probably contributed to the recent increase in the circulation of this virus, that has become the most widespread mosquito-borne flavivirus in recent years [28]. Recently several laboratory studies have been carried out on the potential vector competence of *Cx. pipiens* by experimental infections [35, 38, 39]. Vector competence is a measure of the intrinsic ability of an arthropod to become infected, to support the development or replication of a pathogen, and to transmit it to a vertebrate host.

The aim of this study was to evaluate the vector competence and to determine the potential role in WNV transmission of *Cx. pipiens* mosquitoes collected in different areas of Italy. For this purpose the susceptibility to WNV of *Cx. pipiens* laboratory colonies and field populations was assessed by experimental infections using membrane feeding technique. In addition the progeny from both the first (FGC) and the second (SGC) gonotrophic cycle of the tested populations was analysed in order to assess the possible virus overwintering and amplification maintenance by vertical transmission.

## Methods

### Ethics statement

No specific permissions are required for the field activities which do not involve endangered or protected species. The field sites are not privately-owned or protected properties. The Istituto Superiore di Sanità (ISS) is the National Health Institute of Italy and represents the technical-scientific arm of the Ministry of Health (MoH). All our operative procedures and scientific protocols are previously authorized by the MoH. In this frame, it is involved in vector control activities which authorize it to operate without any specific permission of access to breeding sites and mosquito collections. Moreover the activities reported in the paper were carried out within the 7th Framework Program: European West Nile collaborative research project (Grant agreement n. 261391), approved by European and Italian scientific Committees. This study was carried out in accordance with the recommendations in the Animal Experimentation protocol. All mosquitoes used in our experiments were sacrificed by freezing at the end of the study. Rabbits and quails, used in this study, were not sacrificed. They were from the animal facility of the ISS, where they were maintained according to the Italian and European rules on Laboratory animal care. Blood was collected from the ear vein of the rabbit according to the European legislation for the care and the use of laboratory animals. This is not a part of veterinary care routine but only a blood sample for the infection experiments. At the time, when experiments were performed, the use of laboratory animals in Italy was regulated by legislative decree no. 116/92, which implemented the European Directive 86/609/EEC on laboratory animal protection. In accordance with this legislation the presence and approval of an Ethical Committee is not required; however local welfare veterinarians had the same functions as IACUCs. In particular, at ISS, the veterinarians working for the Service for Biotechnology and Animal Welfare performed the functions of local IACUC; they approved animal research protocols and they verified that the guidelines of legislative decree no. 116/92 on animal welfare were strictly and constantly implemented. All efforts were made to minimize animal suffering. Pig intestine epithelium, used for the experimental infections, is a commercial fresh product, very common in Italy (used for making sausages); it can be found in every butchery or supermarket. We just rinsed it with a saline solution and cut a square portion before use. According to European regulations, manipulations of pathogens belonging to the group 3 (WNV) were carried out in biosafety level 3 (BSL3) facilities. The viral stocks, obtained from human specimens after several passages on Vero cells, are used routinely for research studies. Our laboratory, as National Reference Laboratory for Arboviruses, ISS, routinely analyses human specimens for Arbovirus diagnosis, within the National epidemiologic surveillance activity “Surveillance of human cases of vector-borne diseases with particular reference to Chikungunya, Dengue, and West Nile Disease-2013” (Ministry of Health, DGPRE 0013699-P-14 June 2013 I.4.c.a.9/2011/24). All patient data is anonymized and a written informed consent has been obtained from all participants and/or parents of participants.

### Culex pipiens population features

**Fig. 1:**
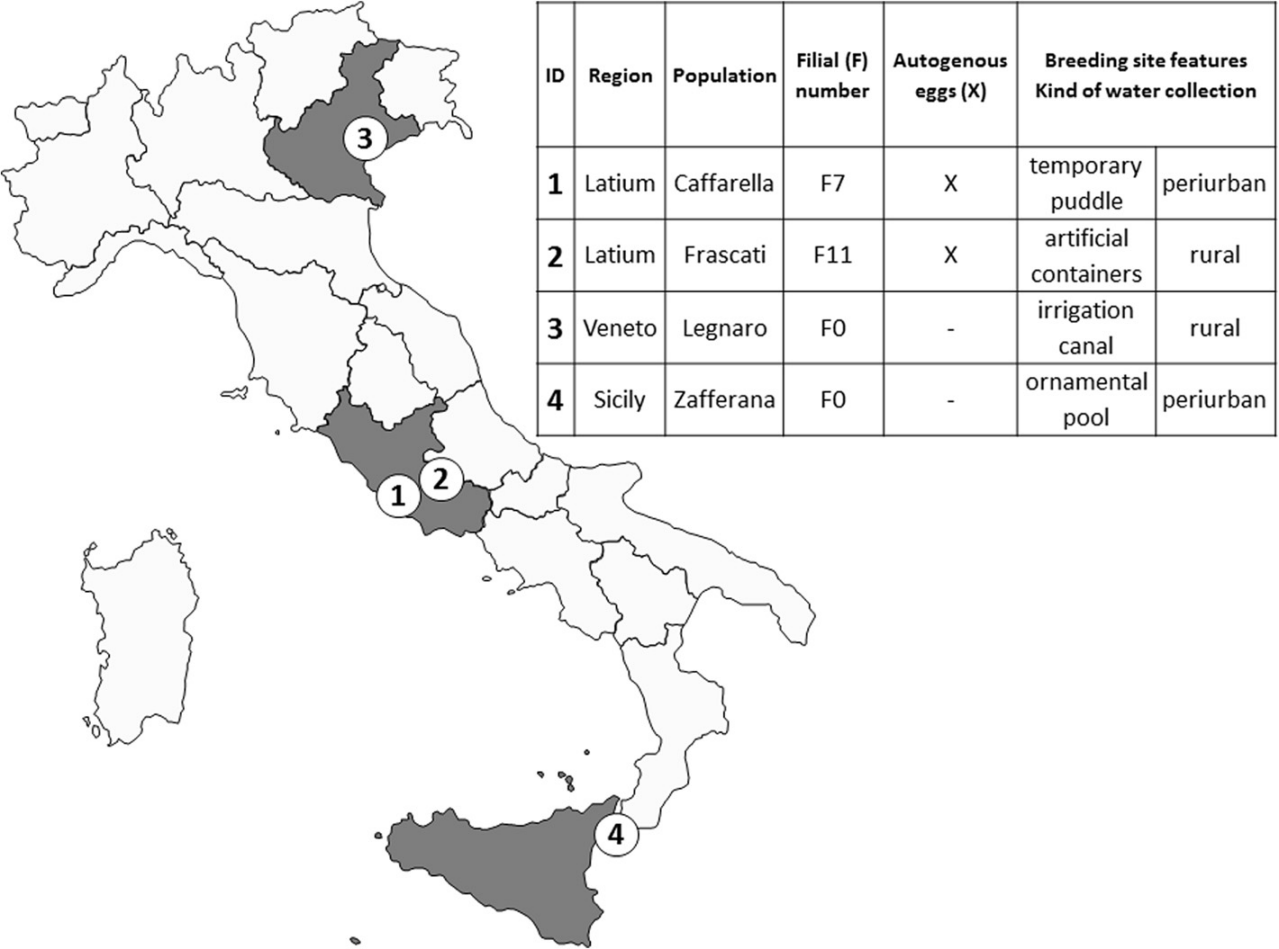
*Cx. pipiens* populations description. Locations and main biological characteristics of the Italian *Cx. pipiens* populations experimentally infected with WNV

For the experimental infections, four Italian *Cx. pipiens* populations were used: two laboratory colonies recently established and two wild populations. Of the two laboratory colonies, one originated from a population collected in a rural site in the municipality of Frascati, (Rome Province, Lazio Region. Geographical coordinates: 41°48’22.33“N 12°40’49.55“E) and the other one originated from an urban park of Rome (Caffarella, Geographical coordinates: 41°51’49.76“N 12°31’10.38“E). Both were reared for several filial generations (F11 for Frascati colony and F7 for Caffarella colony) in the Insectarium of Infectious, Parasitic and Immune-Mediated Diseases Department of Istituto Superiore di Sanità (Rome, Italy). The two rural wild populations were collected at larval stage in the municipalities of Legnaro (Padua Province, Veneto Region. Geographical coordinates: 45°20’12.33“N 11°57’51.51“E) and Zafferana (Catania Province, Sicily Region. Geographical coordinates: 37°41’23.08“N 15° 6’18.98“E) and maintained in Insectarium until adulthood. Collection sites and the main biological characteristics of tested mosquito populations are reported in Fig. 1. In order to allow mating, male and female mosquitoes were bred in the same cage (45×45×45cm) and maintained with sucrose solution (10 %) in climatic chambers under the following environmental parameters: 28 ± 1 °C, a relative humidity (RH) of 70 %, and a light/dark of 16/8 h. Larvae and pupae were reared in a 3 % solution of sodium chloride in distilled water and fed with fish flakes until adulthood. To obtain oviposition, mosquito females were fed on membrane feeding apparatus or on quail for about an hour. At laboratory conditions, autogeny was ascertained only in Frascati and Caffarella colonies, whereas females of the filial generation of Legnaro and Zafferana populations did not lay eggs without a blood meal (Fig. 1).

Before starting the experimental infections, the four *Cx. pipiens* populations were tested to exclude the presence of WN, Chikungunya and Dengue viruses. A representative number of specimens (more than 100) of each populations was analysed as pools of 20 individuals by using both quantitative Real Time PCR (qRT-PCR), and isolation on VERO cells.

### Virus

WNV strain Ma V3, belonging to lineage 1, isolated on VERO cells from the cerebrospinal fluid (CSF) of a patient from Sardinia outbreak in 2011 [25] was used for the experimental infections. The WNV stocks were obtained by propagation on VERO cells and then stored at - 80 °C in aliquots until use. The viral titre of the stocks was 8.43 log_10_ Plaque Forming Units/mL (PFU/mL).

### Experimental infections

To standardize the experimental infection technique, different conditions in the feeding system (membrane system, blood, humidity and room temperature) were tested, using Frascati as reference population, the most adapted to laboratory conditions. The experimental conditions showing the highest efficiency in mosquito feeding were selected and used for all infection experiments.

Infection experiments of the four populations were performed, at the same time, in BSL3 cabinet at room temperature of 28 °C and about 70 % RH. The infectious blood meal was composed of 2/3 rabbit blood with EDTA (Ethylenediaminetetraacetic acid) and 1/3 viral seed, with a final concentration of 7.97 log_10_ PFU/mL.

Female mosquitoes, 8–12 days old, were allowed to feed for 60 min through a pig intestine membrane covering the base of a glass feeder containing the blood-virus mixture, maintained at 37 °C by a warm water circulation system.

After the infectious blood meal, engorged females were selected, transferred to cages and maintained with 10 % sucrose in a climatic chamber at 28 ± 1 °C and 70 % RH.

In order to determine the length of viral extrinsic incubation period, fed females were monitored for 32 days after the infected blood meal. Collections of fed females were carried out at 0–6-9–14-21–28-32 days post-infection (p.i.) and at each time point 5–10 specimens were dissected to examine the viral content in body, legs plus wings and saliva. After chilling, wings and legs were removed from each mosquito to allow for saliva collection. For saliva collection the proboscis was inserted into a quartz capillary filled with 5 μl of Fetal Bovine Serum (FBS) for saliva collection. The mosquitoes were induced to salivate applying on the thorax 2 μl of 1 % pilocarpine in Phosphate buffered saline (PBS) [40]. After 30 min, medium containing the saliva was collected into 500 μl of mosquito diluent (MD) consisting of PBS, 20 % heat-inactivated FBS, 1 % penicillin/streptomycin/amphotericin B mix (Invitrogen, GIBCO). Body, legs plus wings and saliva were stored at -80 °C until processing.

### Cx. pipiens offspring

In order to detect a potential vertical transmission of the WNV, a sample of potentially infected females were allowed to lay eggs. Larvae from the first gonotrophic cycle (FGC) were reared up to adulthood in the climatic chamber. Samples of adults (separated by sex) were collected from the early (4^th^ day p.i.) and late (8^th^ - 10^th^ day p.i.) ovipositions. A second uninfected blood meal was provided to the remaining females at 14^th^ day p.i. and offspring from the second gonotrophic cycle (SGC) were also reared and the adult mosquitoes collected. All samples from FGC and SGC were stored at - 80 °C and processed as pools of 5–20 specimens.

### Real time PCR and viral titration

WNV titre of infected mosquitoes was evaluated both by qRT-PCR and titration by PFU/mL on VERO cells.

For each mosquito, body and legs plus wings were homogenized separately, suspended in 1 mL of MD and centrifuged at 3000 × g for 30 min. The two supernatants obtained and the saliva were used for RNA extraction by using the QIAamp viral RNA kit (Qiagen Inc., Valencia, CA, USA) and for VERO cells inoculation. The qRT-PCR was performed by using WNV TaqMan primers and probe [41]. Briefly, 7 μl of RNA was combined with 20 pmol of each primer and 4 pmol of the FAM- and TAMRA-labelled probe in a 20 μl total reaction volume by using the RNA Virus Master Roche (Roche Diagnostics, Basel, CH). The samples obtained were amplified in an CFX96 Touch™ Real-Time PCR Detection (Bio-Rad), with the following cycling times and temperatures: 1 cycle of retro transcription at 50 °C for 10 min, 1 cycle at 94 °C for 3 min and 45 cycles of 95 °C for 1 s, 60 °C for 20 s (acquisition mode: single), and 72 °C for 1 s (Ramp Rate: 2 °C/s).

Quantification of WNV in RNA samples was determined via qRT-PCR with specifications described previously to estimate plaque forming unit equivalents (PFUeq) [41, 42]. Crossing points values were compared to standard curve based on data acquired from 10-fold serial dilutions of virus stocks with estimated concentration by titration on VERO cells and expressed as log_10_ PFU/mL [42].

The viral titration was performed on six-well plates containing confluent monolayers of VERO cells infected with serial 10-fold dilutions of body and legs plus wings supernatants and of saliva. Cells were incubated at 37 °C for five days under an overlay consisting of Dulbecco’s MEM (DMEM), 2 % FBS, 1 % antibiotic-antimycotic mix (Invitrogen, Gibco) and 2 % tragacant gum (Sigma Aldrich). The plaques were counted after staining them with a solution of crystal violet (0.2 % in 10 % formaldehyde and 20 % ethanol).

### Vector competence indexes

The vector competence of all mosquito populations was assessed by calculating the Infection Rate (IR), Dissemination Rate (DR) and Transmission Rate (TR). Mosquito bodies were analysed in order to evaluate the IR, calculated as the number of WNV positive bodies with respect to the total number of fed females. Legs plus wings were tested to assess the DR, calculated as the number of the specimens with WNV positive legs plus wings out of the number of specimens with WNV positive bodies. The saliva of the potentially infected females was processed to assess the TR, defined as the number of WNV positive saliva out of the number of specimens with WNV positive bodies. The potential vector competence was also expressed as Population Transmission Rate (PTR) calculated as the number of specimens with WNV positive saliva with respect to the total number of fed mosquitoes [42, 43].

### Statistical analysis

Pearson’s Chi-squared test with Yates’ continuity correction was used to determine significant differences (*p* < 0.05) in IR, DR, TR and PTR among the four *Cx. pipiens* populations, and the Kruskall-Wallis test to compare the viral means titres. Statistical analyses were performed using the Stata®13 software (StartCorp LP, Texas, USA).

## Results

### WNV replication dynamics in analysed mosquito populations

**Fig. 2:**
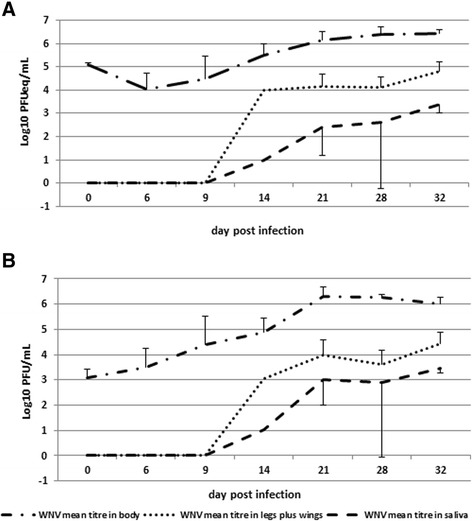
WNV replication in *Cx. pipiens* population collected from Frascati. Comparison of WNV mean titre in infected females of *Cx. pipiens* from Frascati calculated both by qRT-PCR (**a**) and by titration on VERO cells (**b**). *Cx. pipiens* females were exposed to an infectious blood-meal containing 7.97 PFU/mL of WNV; samples of 5-10 fed females were collected at different days post infection and individually analysed for the presence of WNV in body, legs plus wings and in saliva

*Cx. pipiens* specimens from Frascati were first analysed to assess the viral titres in the bodies, legs plus wings and saliva both by qRT-PCR (Fig. 2a) and by titration on VERO cells (Fig. 2b). The qRT-PCR analysis of the mosquito bodies showed mean viral titre of 5.1 ± 0.06 log_10_ (mean ± ΔS) PFUeq/mL, at day 0 p.i. The titres increased reaching a value of 6.43 ± 0.17 log_10_ PFUeq/mL at 32^nd^ day p.i. The viral presence in the legs plus wings and in the saliva was detected starting from 14^th^ day p.i. with a titre of 3.98 and 1.00 log_10_ PFUeq/mL, respectively. Higher values of viral titre in legs plus wings were found in all subsequent collection times peaking a value of 4.82 ± 0.39 log_10_ PFUeq/mL at 32^nd^ day p.i. Also in the saliva the WNV titres increased with values of 2.39 ± 1.21, 2.60 ± 2.84 and 3.40 ± 0.40 log_10_ PFUeq/mL at day 21^st^, 28^th^ and 32^nd^ p.i., respectively (Fig. 2a).

The trend of the mean viral titres, calculated by plaque titration, was very similar to that obtained by qRT-PCR (Fig. 2b). Indeed the mean WNV titre in the bodies increased progressively reaching a value of 6.02 ± 0.26 log_10_ PFU/mL at the 32^nd^ day p.i. The presence of the virus in legs plus wings and saliva was also detected starting from day 14^th^ p.i. with a titre of 3.06 and 1.02 log_10_ PFU/mL, respectively. The viral titres increased in these two compartments up to 32^nd^ day p.i. with values of 4.43 ± 0.46 log_10_ PFU/mL in the legs plus wings and 3.45 ± 0.17 log_10_ PFU/mL in the saliva (Fig. 2b).

**Fig. 3:**
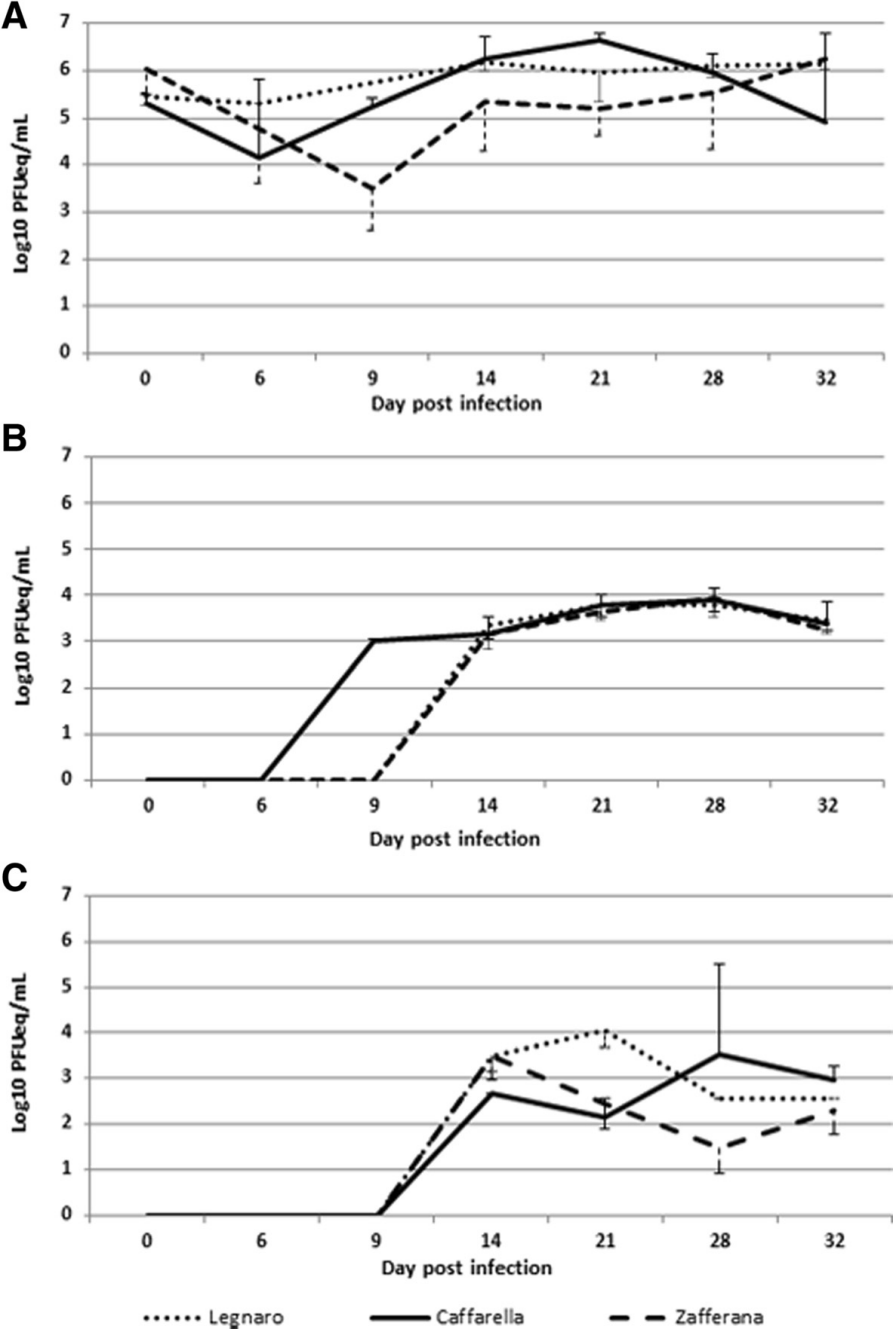
WNV replication in body (**a**), legs plus wings (**b**) and saliva (**c**) in *Cx. pipiens* populations from Legnaro, Caffarella and Zafferana areas. The viral mean titre was calculated by qRT-PCR and expressed in PFUeq/mL. *Cx. pipiens* females were exposed to the WNV infectious blood-meal and samples of fed specimens were collected and analysed for the presence of WNV in body, legs plus wings and in saliva

Due to good correlation of the results obtained by qRT-PCR and titration in VERO cells, the analysis of *Cx. pipiens* populations from Legnaro, Caffarella and Zafferana was performed using qRT-PCR method (Fig. 3). As observed in Frascati colony, WNV was able to infect the mosquitoes collected from Legnaro, Caffarella and Zafferana, to replicate in their body, to disseminate in wings and legs, and to be excreted with saliva. At time 0, the viral titre in the body of Legnaro, Caffarella and Zafferana mosquitoes was of 5.44 ± 0.15, 5.31 ± 0.19 and 6.04 ± 0.52 log_10_ PFUeq/mL, respectively. After an expected initial decrease due to the digestion of the blood meal, viral titres increased gradually during the first days in all mosquito populations. As Frascati colony, the viral growth was continuous in Legnaro and Zafferana, reaching values of 6.16 ± 0.12 and 6.25 log_10_ PFUeq/mL, respectively, at 32^nd^ day p.i., whereas the trend was inverted in Caffarella, peaking at 21^st^ day p.i. (6.65 ± 0.16 log_10_ PFUeq/mL) and decreasing at 32^nd^ day p.i. (4.92 ± 1.89 log_10_ PFUeq/mL) (Fig. 3a). The viral dissemination in Legnaro and Zafferana populations started on 14^th^ day p.i. with a viral titre of 3.33 ± 0.50 and 3.18 ± 0.14 log_10_ PFUeq/mL, respectively, remaining rather constant in subsequent collection times. Otherwise in Caffarella population the dissemination began on the 9^th^ day p.i. (3.02 ± 0.03 log_10_ PFUeq/mL) (Fig. 3b). WNV in the saliva was detected from 14^th^ day p.i. with a viral titre of 3.49 ± 0.34, 2.69 and 3.51 log_10_ PFUeq/mL in Legnaro, Caffarella and Zafferana (Fig. 3c). For Legnaro population, due to lower feeding efficiency on membrane apparatus, the mosquito collection at day 9^th^ p.i. was not carried out. The infectiousness of the viral particles in the saliva of all tested mosquitoes (WNV positive) was demonstrated by viral growth on VERO cells (data not shown).

Our results highlighted that in all *Cx. pipiens* populations, WNV was able to infect mosquitoes and replicate over time, disseminating in wings and legs and reaching the salivary glands.

### WNV vector competence analysis

The cumulative IR, DR, TR and PTR were assessed from 6^th^ day to 32^th^ day p.i. As shown in Table 1 the Legnaro population showed IR (90 %) and DR (100 %) values significantly higher if compared to the other *Cx. pipiens* populations (*p =* 0.037 and *p =* 0.001, respectively). The lower IR and DR values were observed for Zafferana population (55 % and 50 % respectively), while intermediate values were estimated for Frascati (75 % and 54 % respectively) and Caffarella colonies (63 % and 68 % respectively). Out of the total of infected mosquitoes, 37 % of Legnaro and Zafferana, 47 % of Caffarella and 42 % of Frascati were able to excrete WNV by saliva (*p* = 0.915). The highest PTR value was recorded in Legnaro population (33 %) while values of 31 %, 30 %, and 21 % were found in Frascati, Caffarella, and Zafferana populations, respectively (*p* = 0.758) (Table 1).

**Table. 1:** Vector competence indexes for WNV of four Italian populations of *Cx. pipiens*

	Frascati	Legnaro	Caffarella	Zafferana	*Chi* ^*2*^ Test
IR	75 %	90 %	63 %	55 %	*p =* 0.037
(bodies WNV+/tested)	(24/32)	(19/21)	(19/30)	(16/29)	
DR	54 %	100 %	68 %	50 %	*p =* 0.001
(legs plus wings WNV+/bodies WNV+)	(13/24)	(19/19)	(13/19)	(8/16)	
TR	42 %	37 %	47 %	37 %	*p =* 0.915
(saliva WNV+/bodies WNV+)	(10/24)	(7/19)	(9/19)	(6/16)	
PTR	31 %	33 %	30 %	21 %	*p =* 0.758
(saliva WNV+/tested)	(10/32)	(7/21)	(9/30)	(6/29)	

The IR, DR, TR and PTR of analysed *Cx. pipiens* populations were calculated from day 6^th^ to 32^nd^ post infection as following reported: IR (Infection Rate) corresponding to the proportion of mosquitoes with WNV positive body among the total number of fed mosquitoes; DR (Dissemination Rate) corresponding to the proportion of mosquitoes with infected legs plus wings with respect to the number of mosquitoes with infected body; TR (Transmission Rate) corresponding to the proportion of mosquitoes with infected saliva with respect to the number of mosquitoes with infected body; PTR (Population Transmission Rate) corresponding to the proportion of mosquitoes with infected saliva among the total number of fed mosquitoes

### Offspring analysis

In order to detect a possible vertical transmission of WNV in mosquitoes, a total of 377 adult specimens from both the FGC and SGC of the four *Cx. pipiens* populations were analysed in pools. No evidence of vertical transmission was detected, with all 245 male and 132 female progeny testing negative for WNV.

## Discussion

Although WNV has been steadily circulating since 2008 in some regions of Northeastern Italy, and sporadically in other areas of the Country, up to now, no study of vector competence on *Cx. pipiens* Italian populations has been carried out. According to the literature, *Cx. pipiens* is the most common mosquito in many European countries and is also considered to be the primary vector for WNV [21, 28, 31].

In this study, we evaluated the susceptibility for WNV infection, dissemination, and transmission of four *Cx. pipiens* populations originating from North, Central and South/Island areas of Italy, as representative of the whole Country. To our knowledge, this is the first description of the time course study of WNV growth in Italian populations of *Cx. pipiens.* All of the mosquito populations tested were susceptible to infection and the viral growth titres were not significantly different (Figs. 2 and 3). *Cx. pipiens* mosquitoes, both wild (Legnaro and Zafferana) and reared in Insectarium for several generations (Frascati F11 and Caffarella F7), showed a low barrier to midgut infection and WNV was able to escape from the midgut to legs and wings (cumulative IR and DR ≥50 %). IR and DR values of the wild Legnaro population (Table 1), coming from an Italian WNV endemic area, were significantly higher compared to the other populations (*p =* 0.037 and *p =* 0.001, respectively). The cumulative TR of the tested populations showed values ranging from 37–47 % (differences not statistically significant, *p =* 0.915), suggesting the capacity of WNV to reach the salivary glands in all four populations. In addition, the viral titres in the saliva, estimated as genome equivalents, showed no remarkable differences among the four populations, exhibiting values similar to those observed in the literature (2–4 log_10_ PFU/mL) and thought sufficient to successfully infect a potential host [39, 44, 45]. In accordance with the cumulative TR values, the PTRs obtained, measuring the real potential susceptibility of a mosquito population to be infected, confirmed the comparable vector competence of the four Italian *Cx. pipiens* populations (values ranging from 21–33 %, *p =* 0.758).

Generally, the genetic origin (i.e. the degree of anthropophily) and local environmental components (i.e. climate, host seeking, accessibility to humans) represent factors influencing the real efficiency of a vector species (vectorial capacity) [46–48]. The experimental infections, performed under controlled conditions, eliminate or normalize the influence of the extrinsic factors above mentioned. On these bases, eventual differences in the vector competence among mosquito populations of the same species should be reasonably attributed mainly to intrinsic factors. Actually, the question whether the efficiency of a mosquito species to be a good vector may depend on a genetic trait only, is still the object of discussion. Different studies led to hypothesize that the *molestus* form, or even the hybrid ones, may be more competent to transmit WNV [33, 34]. In our study, we assumed a *pipiens* characterization for both the Northern and Southern populations due to the absence of autogenous females. On the contrary, the *molestus* component was certainly present in the other two populations from Central Italy, as displayed already from the first generation with the autogenous ovipositions. Indeed, our results demonstrated that the *Cx. pipiens* populations, even if potentially different in their genetic traits, showed similar capacity to be infected and potentiality to transmit the virus. In addition although different levels of susceptibility to WNV infection have been documented in *Cx. pipiens* populations collected from different areas of California [46, 47], our populations, collected in different areas of Italy, showed a similar vector competence. Despite these results, recurring outbreaks occur in Northeastern Italy but occasionally also in other areas of the Country. These differences in the actual circulation of WNV in Italy seem to depend on a complex of extrinsic factors rather than simply on different intrinsic traits within *Cx. pipiens* populations. Recently Mulatti et al. highlighted how the mosquito density, in combination with key environmental factors, is relevant for a robust prediction of *Cx. pipiens* population expansion and WNV transmission risk [49]. Therefore, the assessment of the genetic characterization of the Italian *Cx. pipiens* populations tested, and the role of the local environmental factors, is in progress in order to better understand of the vectorial capacity of this species.

The analysis of the adults of the FGC and SGC offspring of the four populations didn’t show any evidence of WNV transmission to progeny. However, even if the number of offspring analysed in our study may be considered low (245 males and 132 females), our findings seem to suggest the hypothesis that the vertical transmission would represent a sporadic event not strictly needed for the survival of WNV in the temperate areas of Europe [21]. In addition, very low rates of vertical transmission have been reported also in some WNV experimental infections for North American *Cx. pipiens* populations [50, 51]. Moreover, even if Monaco et al., on the basis of epidemiological studies in North East areas of Italy, strongly suggested the possibility of the WNV overwintering [22], up to now no vertical transmission has been demonstrated in mosquitoes field collected in Italy.

## Conclusion

The results of our study indicate that *Cx. pipiens* Italian populations exhibited a good vector competence for Italian WNV lineage 1 strain, addressing an important issue in the complex WNV-vector relationships. Insights on vector competence, distribution, seasonal density, and natural feeding patterns of *Cx. pipiens* can help to improve WNV surveillance in Italy, since current knowledge does not allow yet to predict where and when WNV outbreaks will occur.
